# AGE-INCLUSIVE PRINCIPLES ON CAMPUS: EMBRACING DIVERSITY ACROSS THE LIFESPAN

**DOI:** 10.1093/geroni/igac059.2758

**Published:** 2022-12-20

**Authors:** Kelly Munly, Lauren Jacobson

**Affiliations:** Penn State - Altoona, Altoona, Pennsylvania, United States; Penn State Altoona, Altoona, Pennsylvania, United States

## Abstract

In this poster, researchers based at a Mid-Atlantic university campus provide an understanding of their evaluative steps informing an adaptation of the Diversity Circles program to include a multigenerational component, supporting diversity across the lifespan. Project methods and analysis have been informed by critical theoretical frameworks, including feminist gerontology, that illuminate the invisibility of age, even in the context of intersectional work. Pilot feedback from five participants from a condensed program in an Adult Development and Aging course informed the interview approach. Post-program semi-structured interviews, with program participants, including students and older adults (n=7), and community stakeholders (n=18), provided feedback on diversity needs at the campus and in the surrounding community, as well as on program content and experience and opportunity for further curriculum integration of concepts of age-friendliness, ageism, and age-awareness. Stakeholders interviewed included community practicum liaisons, university advising and student affairs staff, faculty and staff previously engaging in diversity-related activities, university administrators, university personnel attending to enrollment matters, and staff and faculty interested in student-centered curriculum design. Semi-structured interviews were chosen for data collection because of their capacity to provide saturated data from a small, purposeful sample. Focused codes emergent from the interviews included both a) suggestions for curriculum adaptation and modification as well as the value of existing content and b) issues of age-friendliness and ageism more generally. The research team looks toward incorporating suggestions within their findings in an expansion of the program on their campus, and disseminating findings for the benefit of other campuses’ programs.

